# Exercise duration-matched interval and continuous sprint cycling induce similar increases in AMPK phosphorylation, PGC-1α and VEGF mRNA expression in trained individuals

**DOI:** 10.1007/s00421-016-3402-2

**Published:** 2016-06-01

**Authors:** Conor W. Taylor, Stephen A. Ingham, Julie E. A. Hunt, Neil R. W. Martin, Jamie S. M. Pringle, Richard A. Ferguson

**Affiliations:** School of Sport, Exercise and Health Sciences, Loughborough University, Loughborough, Leicestershire LE11 3TU UK; English Institute of Sport, EIS Performance Centre, Loughborough University, Loughborough, LE11 3TU UK; English Institute of Sport, Manchester Institute of Health and Performance, 299 Alan Turing Way, Manchester, M11 3BS UK; Faculty of Health and Medical Sciences, School of Biosciences and Medicine, University of Surrey, Guildford, GU2 7YW UK; British Athletics, National Performance Institute, Loughborough University, Loughborough, LE11 3TU UK

**Keywords:** Angiogenesis, HIF-1α, High-intensity training, Mitochondrial biogenesis, Sprint interval exercise

## Abstract

**Purpose:**

The effects of low-volume interval and continuous ‘all-out’ cycling, matched for total exercise duration, on mitochondrial and angiogenic cell signalling was investigated in trained individuals.

**Methods:**

In a repeated measures design, 8 trained males ($\dot{V}{\text{O}}_{{2{\text{peak}}}}$, 57 ± 7 ml kg^−1^ min^−1^) performed two cycling exercise protocols; interval (INT, 4 × 30 s maximal sprints interspersed by 4 min passive recovery) or continuous (CON, 2 min continuous maximal sprint). Muscle biopsies were obtained before, immediately after and 3 h post-exercise.

**Results:**

Total work was 53 % greater (*P* = 0.01) in INT compared to CON (71.2 ± 7.3 vs. 46.3 ± 2.7 kJ, respectively). Phosphorylation of AMPK^Thr172^ increased by a similar magnitude (*P* = 0.347) immediately post INT and CON (1.6 ± 0.2 and 1.3 ± 0.3 fold, respectively; *P* = 0.011), before returning to resting values at 3 h post-exercise. mRNA expression of PGC-1α (7.1 ± 2.1 vs. 5.5 ± 1.8 fold; *P* = 0.007), VEGF (3.5 ± 1.2 vs. 4.3 ± 1.8 fold; *P* = 0.02) and HIF-1α (2.0 ± 0.5 vs. 1.5 ± 0.3 fold; *P* = 0.04) increased at 3 h post-exercise in response to INT and CON, respectively; the magnitude of which were not different between protocols.

**Conclusions:**

Despite differences in total work done, low-volume INT and CON ‘all-out’ cycling, matched for exercise duration, provides a similar stimulus for the induction of mitochondrial and angiogenic cell signalling pathways in trained skeletal muscle.

## Introduction

Low-volume sprint interval training (SIT), typically comprising 4–6 × 30 s ‘all-out’ efforts interspersed by 4 min of recovery, has proven as effective as traditional, high-volume endurance training (e.g. >40 min continuous moderate intensity training) in increasing muscle oxidative potential, buffering capacity, resting muscle glycogen content and muscle capillarity (Gibala et al. 2006; Burgomaster et al. 2008; Cocks et al. 2013). This time-efficient training model presents health benefits for sedentary and clinical populations and a potentially potent stimulus for enhancing athletic performance. Indeed, endurance athletes are typically accustomed to high-volume training methods, and thus, novel work- and time- efficient exercise stimuli to further enhance training adaptation are desirable.

The underlying factors responsible for the comparable skeletal muscle re-modelling and exercise performance after SIT and high-volume endurance training remain unclear (Baar et al. 2002; Pilegaard et al. 2003; Lee-young et al. 2009; Gibala et al. 2009). In addition to differences in the initial fitness levels of the aforementioned study cohorts and differences in total work done, exercise duration and intensity, the interval nature of SIT is a distinguishing factor between the training protocols prescribed. Recent studies (Bartlett et al. 2012; Cochran et al. 2014) have examined the effects of this ‘pulsatile’ exercise pattern on the acute activation of cell signalling cascades that if repeatedly stressed over time, regulate endurance based skeletal muscle adaptations and in particular mitochondrial biogenesis (Perry et al. 2010).

In this regard, homeostatic perturbations within skeletal muscle (e.g. increased AMP/ATP ratios and reductions in muscle glycogen) phosphorylate and activate protein kinases, such as the adenosine monophosphate-activated protein kinase (AMPK). Upon activation, AMPK converges on the cell nucleus and is implicated in the regulation of peroxisome proliferator-activated γ receptor coactivator (PGC-1α) (Jäger et al. 2007; Cantó and Auwerx 2010). Via interactions with downstream transcription factors and nuclear receptors, PGC-1α is considered to play a “master” regulatory role in exercise-induced mitochondrial biogenesis (Puigserver and Spiegelman 2003) and angiogenesis through an interaction with vascular endothelial growth factor (VEGF) (Leick et al. 2009; Chinsomboon et al. 2009; Geng et al. 2010). Indeed, similar increases in AMPK phosphorylation and PGC-1α mRNA expression have been observed after work/duration-matched (~50 min) interval and continuous high-intensity running in recreationally active men (Bartlett et al. 2012). More recently, a study by Cochran and colleagues (Cochran et al. 2014) observed similar increases in acetyl-CoA carboxylase (ACC) phosphorylation (a surrogate marker of AMPK activation) and PGC-1α mRNA expression in response to work-matched SIT and maximal continuous (~4 min) cycling. However, whilst acute SIT has previously been shown to increase upstream genetic markers of mitochondrial biogenesis in both recreationally active individuals (Gibala et al. 2009) and ‘elite level’ cyclists (Psilander et al. 2010), its effects on regulators of exercise-induced angiogenesis (e.g. VEGF, HIF-1α, eNOS, MMP-9) have not been reported in untrained or trained muscle.

Since work done and the pattern of exercise (i.e. interval vs. continuous) have previously been demonstrated to have little effect on acute cell signalling responses, it was hypothesised that despite differing in total work done, brief ‘all-out’ cycling, be it repeated intervals or a continuous single effort, would induce similar increases in cell signalling cascades linked to mitochondrial biogenesis and angiogenesis in trained skeletal muscle.

## Methods

### Participants

Eight healthy males (mean ± SD: age, 30 ± 5 yr; stature, 180 ± 9 cm; body mass, 79 ± 11 kg; PPO, 16 ± 2 W.kg; $\dot{V}{\text{O}}_{{2{\text{peak}}}}$, 4.5 ± 0.5 L min^−1^; 57 ± 7 ml kg^−1^ min^−1^; MAP, 347 ± 27 W) volunteered to take part in the investigation. All were involved in cycling activities three to six times per week and on at least one occasion per week also performed resistance training consisting of lower body bilateral compound exercises (e.g. squats, Olympic lifting). Although all participants had previous experience of undertaking high-intensity training, none were engaging in this type of training during the study period. Participants completed a medical questionnaire and biopsy screening document prior to participation to mitigate for maximal exercise and biopsy contraindications. Participants did not have a history of neuromuscular, haematological or musculoskeletal abnormalities or were using pharmacological treatments during the study period. The participants were fully informed of the purposes, risks and discomforts associated with the investigation before providing written consent. The investigation conformed to current local guidelines and the Declaration of Helsinki and was approved by the Ethics Approval (Human Participants) Sub-Committee of the Loughborough University Ethical Advisory Committee.

### Experimental design

In a fully randomised, repeated measures crossover design, participants performed ‘all-out’ interval (INT) or continuous (CON) cycling protocols, which were separated by 14–21 days. Muscle biopsies were obtained before, immediately and 3 h post-exercise. Participants recorded all food consumed and physical activity performed during the 24 h prior to their first experimental trial and were instructed to replicate these dietary and activity patterns prior to their second experimental trial as well as refrain from ingesting alcohol and caffeine containing substances during the 48 h preceding each trial. Participants were instructed to arrive at the laboratory by identical means of travel for both experimental trials. All arrived by car and made a short walk to the ground floor laboratory in which all testing took place (i.e. very minimal work was performed in getting to the laboratory). All cycle protocols were performed on an SRM ergometer (Schroberer Rad McBtechink, Weldorf, Germany) calibrated according to the manufacturer guidelines.

### Preliminary testing

Two weeks prior to the first experimental trial, participants were reported to the laboratory on three occasions, separated by at least 3 days to complete preliminary measurements and protocol familiarisation. During the first visit optimal pedal cadence for peak power output (PPO) was determined by performing maximal sprints (<12 s) at multiple fixed cadences. The optimal pedal cadence, defined as the zenith of the cadence-peak power relationship, was subsequently used as the fixed (isokinetic) cadence for the subsequent experimental trials. A maximal incremental cycle test was also performed during this visit to establish peak oxygen uptake ($\dot{V}{\text{O}}_{{2{\text{peak}}}}$) and maximal aerobic power (MAP) to establish aerobic training status. Following a 5 min warm-up at 120 W, at a freely chosen but constant pedal cadence, work rate was increased by 20 W every 60 s until volitional exhaustion (8–10 min). Pulmonary gas exchange was measured breath by breath throughout exercise (Oxycon Pro, Carefusion, UK) and $\dot{V}{\text{O}}_{{2{\text{peak}}}}$ determined as the highest 30 s recording of $\dot{V}{\text{O}}_{2}$ during the test. MAP was defined as the highest average 60 s power recorded during the test. On the two subsequent visits participants performed familiarisation trials during which they performed the INT and CON protocols. The cycle ergometer saddle and handlebar configuration was consistent for each participant during all exercise testing.

### Experimental protocols

Participants attended the laboratory in the morning (~0800 h) of each experimental trial following an overnight fast. On arrival, participants rested in a supine position for 20 min whilst the muscle biopsy sites were prepared. After a resting muscle biopsy was taken, participants performed a standardised warm-up, consisting of cycling at 120 W for 5 min, immediately after which they performed either the INT or CON cycling trials. The INT protocol consisted of 4 × 30 s ‘all-out’ efforts at the predetermined fixed (isokinetic) pedal cadence. Each bout was interspersed by 4 min of recovery during which participants remained seated on the cycle ergometer and were permitted to cycle at a cadence of ~60 revs min^−1^ against a resistance of <20 W. This resulted in total session duration of 14 min, excluding the 5 min warm-up. The CON protocol consisted of a single 2 min bout of continuous ‘all-out’ cycling at the same isokinetic pedal cadence. To ensure each protocol was performed in an ‘all-out’ manner, participants were verbally instructed and encouraged before commencing and during each protocol to attain PPO within the first few seconds of each exercise bout and apply maximal force through the pedals until the end of each exercise bout. Immediately after both protocols, participants quickly dismounted the ergometer and were helped onto an adjacent couch where the immediate post-exercise muscle biopsy was taken within 1 min of the cessation of each protocol. During this time, a finger-prick capillary blood sample was obtained every consecutive minute, and immediately analysed for lactate concentration (Biosen HBAC1 Analyser, EKF Diagnostics, UK) until a peak lactate concentration was determined (within 10–15 min). Participants then rested before a final muscle biopsy was taken 3 h post-exercise. Biopsies for a given trial were obtained from the same leg through separate incisions ~3 cm apart. In the subsequent experimental trial, biopsies were obtained from the alternate leg in a randomised, crossover fashion to avoid any potential order bias. The consumption of food was prohibited at all times during each experimental trial. Water was consumed freely but the volume and pattern ingested was noted during the first trial and replicated for the subsequent trial. Laboratory conditions remained constant for both experimental trials (19–21 °C, 40–50 % humidity).

Power output during preliminary testing and both experimental protocols were sampled at 0.5 Hz and analysed using SRM software (v6.40.05, Schoberer Rad Messtechnik, Germany) which was calibrated before each trial by recording the zero offset without any force/load on the cranks. Peak power output (PPO) was defined as the highest single power output value recorded during each sprint. Mean power output (MPO) was defined as the average value of all measures of power output throughout each sprint.

### Muscle biopsy sampling and analysis

Muscle biopsies were obtained from the medial portion of the vastus lateralis muscle under local anaesthesia (1 % lidocaine) using the micro-biopsy technique (Acecut 11G Biopsy Needle, TSK). Muscle samples at each time point were obtained through separate incisions with two samples (each ~25 mg) being taken from each incision.

#### Western blot analysis

Approximately 20 mg of frozen muscle was ground to powder under liquid nitrogen using a laboratory grade pestle and mortar before being homogenised in 120 µl of ice cold lysis buffer (25 mM Tris/HCl [pH 7.4], 50 mM NaF, 100 mM NaCl, 5 mM EGTA, 1 mM EDTA, 10 mM Na-Pyrophosphatase, 1 mM Na_3_VO_4_, 0.27 M sucrose, 1 % Triton X-100, 0.1 % 2-mercaptoethanol) supplemented with a Pierce™ Protease Inhibitor Tablet (Thermo Scientific, UK). Homogenates were centrifuged at 13,500*g* for 10 min at 4 °C and the supernatant collected. Protein content of the supernatant was determined using a Pierce™ 660 Protein Assay (Thermo Scientific, UK). Each sample was solubilized for 5 min at 100 °C with an equal volume of sample buffer containing 1 M Tris–HCl (pH 6.8), 8 % glycerol, 10 % sodium dodecyl sulphate, 0.4 % 2-β-mercaptoethanol and 0.05 % bromophenol blue. For each blot a negative control was loaded along with 25 µg of each sample and then separated (~100 V for ~2 h) in Tris–glycine running buffer using self-cast 4 % stacking and 10 % separating polyacrylamide gels. Gels were transferred wet onto nitrocellulose membranes for 2 h at 35 mA in a 1× transfer buffer (0.3 % Tris base, 1.4 % glycine, 20 % methanol). Membranes were then blocked for 1 h at room temperature in Tris-buffered saline (TBST: 0.19 M Tris [pH 7.6], 1.3 M NaCl, 0.1 % Tween-20) with 5 % non-fat blocking grade milk. Membranes were washed for 3 × 5 min in TBST before being incubated overnight at 4 °C with anti-phospho^Thr172^ (cat no. 2531) and anti-total AMPK (cat no. 2532) antibody (both from cell signalling, UK), at a concentration of 1:1000 in 1 × TBST. The following morning, membranes were washed for a further 3 × 5 min in TBST, and subsequently incubated with anti-species horseradish peroxidase-conjugated secondary antibody (Bio-Rad, UK) for 1 h at room temperature. After a further 3 × 5 min washes in TBST, membranes were saturated in chemiluminescence (SuperSignal, Thermo Fisher Scientific, Rockford, IL, USA) for 5 min prior to exposure. Membranes were visualised using image analysis (ChemiDoc™ XRS+, Bio-Rad, Herts, UK), and band densities determined (Quality One 1-D analysis software v 4.6.8, Bio-Rad, Herts, UK). Samples from each participant for both exercise protocols were run on the same gel and all gels were run in duplicate to verify responses. Pre-exercise values of phosphorylation relative to total for each participant were normalised to 1 with post-exercise and 3 h post-exercise values subsequently expressed as fold change relative to pre-exercise values.

#### Real-time RT-PCR

One-step quantitative RT-PCR was used to determine skeletal muscle mRNA levels of genes of interest. Primer sequences (Table 1) were designed by Sigma-Aldrich (Sigma-Aldrich Co. Ltd., Haverhill, UK) ideally with 40–60 % GC content and spanning exon–exon boundaries. Primer specificity was determined by performing BLAST and melt curve analysis at the end of each PCR run. Total RNA was isolated from muscle tissue using TRIzol^®^ according to the manufacturer’s instructions (Life Technologies/Invitrogen, USA). Briefly, once the tissue (~25 mg) was homogenised in TRIzol^®^, chloroform (1:5 v/v) was added followed by RNA precipitation using isopropanol. The resultant RNA pellet was washed in 75 % absolute ethanol and air dried prior to resuspension in 50 µL of 1 mM sodium citrate. RNA concentration (232 ± 73 ng μl^−1^) and purity (260/280: 1.9 ± 0.1) was confirmed using spectrophotometry (Nanodrop) before being stored at -80 ^◦^C for future use. 20 µl PCR reactions were made up as follows in a 96 well plate; 70 ng of RNA in 9.5 µl of nuclease free water, 0.2 µl of Quantifast Reverse Transcriptase mix (Qiagen, Crawley, UK), 0.15 µl of both forward and reverse primers at 100 µM concentrations, and 10 µl of SYBR green mix (Qiagen). All reactions were performed in triplicate. Once PCR plates were prepared, they were transferred to the mx3005p qPCR cycler (Stratagene MX3005P, Agilent Technologies, Berkshire, UK), which was programmed to perform the following steps; 50 °C for 10 min (reverse transcription), followed by a 5 min hold at 95 °C, and then 40 cycles at 95 °C for 10 s and 60 °C for 30 s. Fluorescence was detected at the end of each cycle, and expression levels were determined using the 2^−∆∆*Ct*^ method using RNA polymerase II (RPII) as the reference gene. The mRNA expression was calculated according to Livak and Schmittgen (2001). Post-exercise values are reported as a fold change relative to pre-exercise values for each individual participant as described previously (Pilegaard et al. 2000; Psilander et al. 2010).

**Table 1:** Primers used for real-time RT-PCR analyses

Target gene		Primer sequence	No. in gene bank
VEGF	Forward Reverse	CTGCTCTACCTCCACCAT ATGAACTTCACCACTTCGT	NM_001171630
PGC-lα	Forward Reverse	CCTCTTCAAGATCCTGCTA ACTCTCGCTTCTCATACTC	NM_013261
eNOS	Forward Reverse	CAAGTTGGAATCTCGTGAA TGTGAAGGCTGTAGGTTAT	NM_001160111
MMP-9	Forward Reverse	GGCACCTCTATGGTCCTC AGTAGTGGCCGTAGAAGG	NM_004994
HIF-lα	Forward Reverse	TCACCTGAGCCTAATAGTC AATCTGTGTCCTGAGTAGAA	NM_181054
RPII	Forward Reverse	GAGTCAACGGATTTGGTC GGTGGAATCATATTGGAACAT	NM_000938.1

*VEGF* vascular endothelial growth factor, *PGC-lα* peroxisome proliferator-activated receptor γ coactivator-lα, *eNOS* endothelial nitric oxide synthase, *MMP-9* membrane metalloproteinase-9, *HIF-lα* hypoxic inducible factor-lα, *RPII* RNA polymerase II

### Statistical analysis

Protein phosphorylation and mRNA data were analysed using a two-way ANOVA. Where significant main effects were observed, Bonferroni corrected post hoc t-tests were used to locate differences. Student’s *t* test for paired samples was also used to compare differences in physiological and performance variables between protocols. All data are presented as mean ± SE. Significance was accepted at *P* < 0.05.

## Results

### Physiological and performance variables

**Fig. 1:**
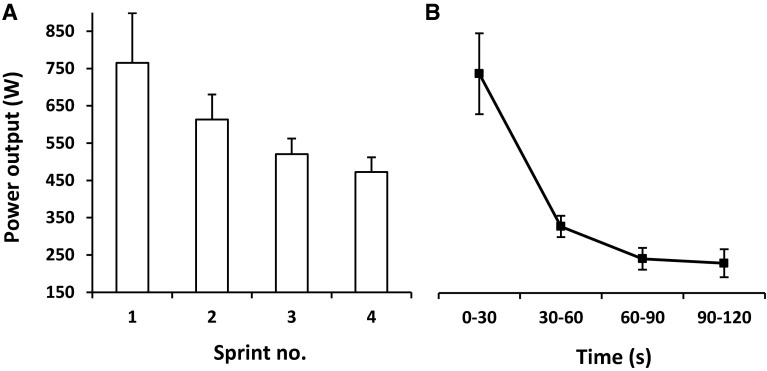
MPO achieved for each of the 4 × 30 s ‘all-out’ sprints during INT (**a**) compared to the MPO achieved in each consecutive 30 s period of the continuous 2 min ‘all-out’ effort in CON (**b**)

The optimal cadence for PPO was 118 ± 5 revs min^−1^. There was no difference (*P* = 0.93) in PPO attained during INT and CON (1217 ± 257 vs. 1215 ± 201 W, respectively). However, there was a greater MPO (Fig. 1) during INT compared to CON (593 ± 61 vs. 386 ± 23 W, *P* = 0.01) resulting in 53 % greater total work done in the INT compared to CON (71.2 ± 7.3 vs. 46.3 ± 2.7 kJ, *P* = 0.01). Peak blood lactate concentration was higher (*P* = 0.04) following INT compared to CON (19.2 ± 1.0 vs. 17.4 ± 2.1 mmol L^−1^, respectively).

### AMPK activation

**Fig. 2:**
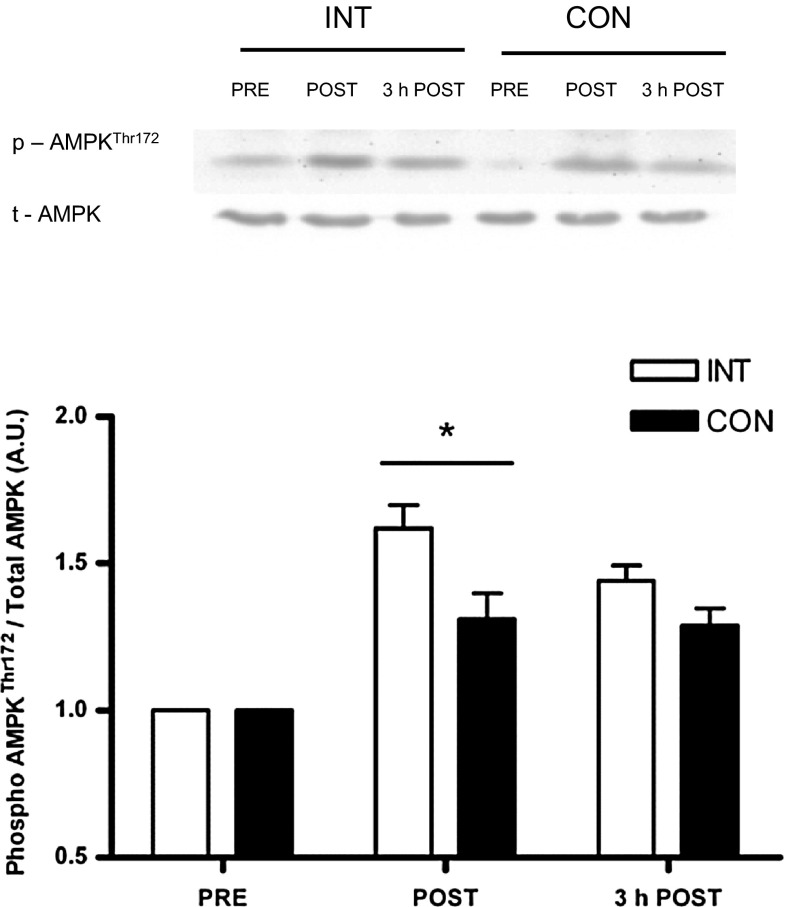
Phosphorylation of AMPK^Thr172^ expressed relative to total AMPK immediately before (PRE), after (POST) and 3 h after (3 h POST) the INT and CON protocols. Representative Western blots above figure. *p* phosphorylated; *t* total. Each participant’s PRE values have been normalised to 1, and thus, POST and 3 h POST values are expressed as fold change relative to PRE values. Values are mean ± SE (*n* = 8). *Significant difference from PRE and 3 h POST (*P* < 0.05)

Phosphorylation of AMPK^Thr172^ increased (*P* = 0.011) 1.6- and 1.3-fold immediately following INT and CON, respectively, before returning to baseline at 3 h post-exercise (Fig. 2). The magnitude of phosphorylation immediately post-exercise was not different between protocols (*P* = 0.347).

### mRNA expression

**Fig. 3:**
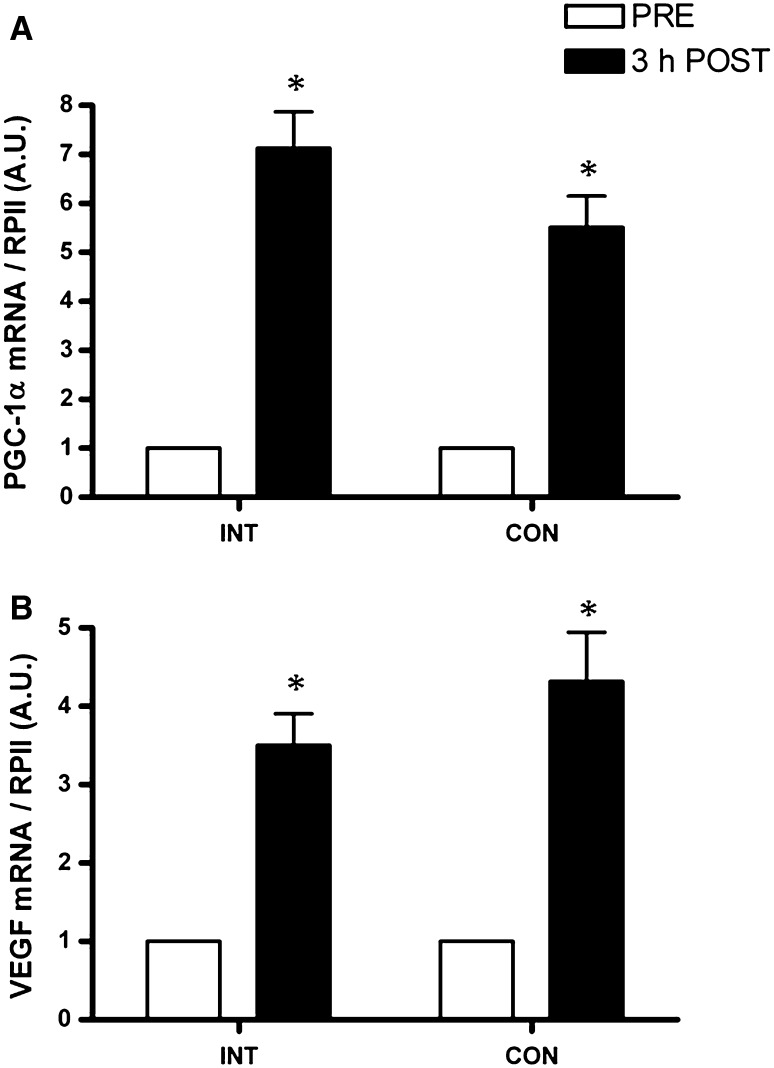
PGC-1α (**a**) and VEGF (**b**) mRNA expression immediately before (*open bars*) and at 3 h after (*closed bars*) the INT and CON protocols normalised to RPII mRNA content and expressed relative to PRE. Values are mean ± SE (*n* = 8). *Significant difference from PRE (*P* < 0.05)

**Fig. 4:**
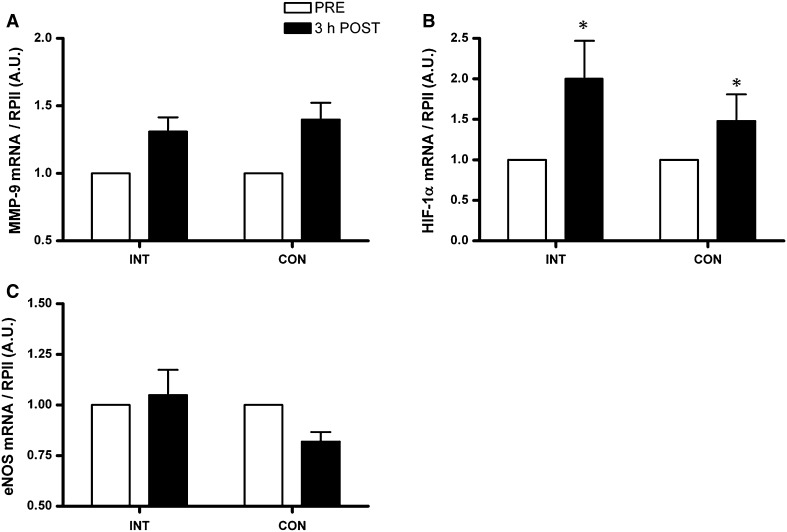
MMP-9 (**a**), HIF-1α (**b**) and eNOS (**c**) mRNA expression immediately before (*open bars*) and at 3 h after (*closed bars*) the INT and CON protocols normalised to RPII mRNA content and expressed relative to PRE. Values are mean ± SE (*n* = 8). *Significant difference from PRE (*P* < 0.05)

There was an increase in mRNA expression of PGC-1α (5.5- vs. 7.0-fold, *P* < 0.01) and VEGF (4.3- vs. 3.5-fold, *P* = 0.02) from pre to 3 h post-exercise in CON and INT, respectively, the magnitude of which were not different between trials (Fig. 3). HIF-1α mRNA expression increased (1.5- vs. 2-fold, *P* = 0.04), and there was a trend for MMP-9 mRNA expression (1.4 vs. 1.3-fold, *P* = 0.06) to increase from pre to 3 h post-exercise in CON and INT, respectively, the magnitude of which were not different between trials (Fig. 4). There was no change in eNOS mRNA expression (*P* = 0.6) in either CON or INT (Fig. 4).

## Discussion

This study has demonstrated that low-volume interval and continuous ‘all-out’ sprint cycling, both totalling 2 min in duration but differing in total work done, induce comparable AMPK phosphorylation and increases in PGC-1α, VEGF and HIF-1α mRNA expression in trained individuals.

The similar increases in AMPK phosphorylation observed immediately after both protocols in the present study are consistent with those previously reported in trained (Yeo et al. 2010; Little et al. 2010) and untrained (Gibala et al. 2009; Little et al. 2011) human muscle after various cycling protocols. The capability of INT and CON to induce comparable acute cell signalling responses to each other, despite 53 % more work being performed in INT, and similar magnitudes of increase to that observed after traditional high-volume endurance training appears related to the ‘all-out’ nature of the exercise. This possibly reflects the rapid and significant depletion of ATP and muscle glycogen content that occurs in all fibre types and in particular type II muscle fibres during ‘all-out’ sprint exercise (Greenhaff et al. 1994; Bogdanis et al. 1996; Hargreaves et al. 1998; Karatzaferi et al. 2001). Indeed, the highly glycolytic type IIX fibres display the greatest basal and exercise-induced increases in AMPK phosphorylation (Lee-young et al. 2009) and the high rates of glycogenolysis observed during this type of exercise (Hargreaves et al. 1998) is likely to play a role given the importance of muscle glycogen in regulating AMPK activity (Wojtaszewski et al. 2003; Yeo et al. 2008; Philp et al. 2012).

In accordance with the role of upstream AMPK activation in regulating PGC-1α (Jäger et al. 2007), the similar fold changes in PGC-1α mRNA observed at 3 h after INT and CON are consistent with magnitudes and time courses previously reported in response to cycling and running in trained (Little et al. 2010; Psilander et al. 2010) and untrained (Gibala et al. 2009; Egan et al. 2010; Bartlett et al. 2012) individuals. Whilst our data supports previous observations (Gibala et al. 2009), we also provide novel data that a single 2 min continuous ‘all-out’ effort provides an equal stimulus for PGC-1α expression. Like AMPK, PGC-1α mRNA abundance is increased in an intensity-dependent manner in the hours after exercise, (Egan et al. 2010; Tobina et al. 2011). Increases in PGC-1α protein content, however, typically occur in the days following exercise (Baar et al. 2002), probably as a result of cumulative transient increases in mRNA transcripts encoding new protein after successive training bouts (Perry et al. 2010). Further research is required to determine whether both protocols used here would result in comparable PGC-1α protein increases after an identical number of successive training sessions in both trained and untrained individuals.

PGC-1α is implicated in a signalling cascade that results in the induction of VEGF (Chinsomboon et al. 2009; Geng et al. 2010), the most important angiogenic growth factor, integral to both longitudinal splitting and sprouting angiogenesis (Olfert et al. 2010; Egginton 2011). It is possible that PGC-1α played a role in increasing the VEGF mRNA expression observed after both ‘all-out’ exercise protocols given the regulation of PGC-1α by post-translational modifications (i.e. phosphorylation and deacetylation) (Puigserver et al. 2001; Jäger et al. 2007). The similar magnitudes of VEGF mRNA increase that we report here are consistent with those observed in the early hours of recovery after acute exercise of varied intensities and durations (Gustafsson et al. 1999; Jensen et al. 2004b; Hoier et al. 2012, 2013b), and to our knowledge are the first reported in response to both interval and continuous ‘all-out’ cycling. Although clearly important, it remains to be fully established whether VEGF mRNA is increased during recovery from exercise primarily to replenish secreted VEGF protein or is in fact vital to trigger VEGF secretion.

Distinct angiogenic phenotypes occur as a result of exposure to different stimuli which are affected by the mode and intensity of exercise (Egginton 2011). HIF-1α is a key regulator of the tissue to hypoxia, and thus, metabolic stress (Semenza et al. 2006; Semenza 2006), during which it is stabilized and functions in regulating angiogenesis through targeted activation of VEGF in human skeletal muscle (Lee et al. 2004; Ameln et al. 2005). Acute exercise results in an increase in skeletal muscle HIF-1α mRNA expression provided that the exercise intensity is sufficient or the tissue is exposed to a hypoxic stimulus (Vogt et al. 2001; Zoll et al. 2006). Thus, the comparable increases in HIF-1α mRNA expression observed in the early recovery after exercise in the present study may tentatively suggest INT and CON provide a similar stimulus for hypoxia-mediated angiogenesis. Neither of the protocols used here increased eNOS mRNA which has been implicated in high-shear stress mediated angiogenesis (Egginton 2009). However, there was a trend (*P* = 0.06) in the present study for comparable increases in MMP-9 mRNA after INT and CON. MMP-9 is activated in response to muscle overload or mechanical stretch during contractions and plays a role in initiating proteolysis of the endothelial cell (EC) basement membrane, thus facilitating EC migration and the formation of new capillaries (Egginton 2009). Increased MMP-9 mRNA expression has previously been observed 2 h into recovery from moderate intensity endurance exercise in healthy human skeletal muscle (Rullman et al. 2007, 2009) and such transcriptional activation appears important in the regulation of MMP-9 activity (Van den Steen et al. 2002).

The present study is not without limitations. Notably, exercise-induced changes in muscle glycogen content, protein expression reflective of mitochondrial biogenesis and angiogenesis, and measures of angiogenic protein secretion into the circulation were not assessed in the present study. Indeed, although previous studies have demonstrated that the acute angiogenic response can help to inform the magnitude of capillary growth with training (Hellsten et al. 2008; Høier et al. 2010; Hoier et al. 2012), temporal factors relating to the timing and number of biopsies, as well as the training status of the examined muscle, need to be considered when predicting chronic adaptations based on transient molecular responses to acute exercise. Intermittent high-intensity exercise has been previously shown to induce greater elevations of angiogenic growth factors than moderate intensity continuous exercise; however, the latter has been demonstrated to result in greater acute elevations in interstitial levels of VEGF (Hoier et al. 2013a). Moreover, whilst increased capillarity has been observed after 4 weeks of high-intensity intermittent knee extensor exercise in young healthy individuals (Jensen et al. 2004a), others have provided data suggesting high-intensity intermittent training, provides a weak stimulus for capillary growth in a similar population (Hoier et al. 2013a). Although it is plausible from our acute data that trained skeletal muscle exposed to repeated bouts of INT and CON over time would promote comparable increases in mitochondrial biogenesis and capillarity further work is required to directly assess this. Finally, future work should also consider whether this 2 min all-out effort repeated over a period of time has the capacity to improve overall fitness. Cochran et al. (2014) have recently demonstrated in untrained individuals that whilst ‘all-out’ SIT and work-matched continuous cycling (~4 min) induce similar acute cell signalling responses, when performed regularly over a 6 week training regime, the continuous protocol did not augment the maximal activity or protein content of mitochondrial markers as has been observed after a similar exposure to SIT in earlier work by this group (Gibala et al. 2006; Burgomaster et al. 2008). Thus, in their relatively untrained cohort, the continuous protocol (which should be noted was not ‘all-out’ in nature as evidenced by the significantly lower mean peak power output attained in this trial in comparison to SIT) was sufficient to increase $\dot{V}{\text{O}}_{{2{\text{peak}}}}$ (Cochran et al. 2014).

In conclusion, low-volume ‘all-out’ sprint interval and continuous cycling, matched for total exercise duration but not work done, provides an equally potent stimulus to activate cell signalling pathways associated with exercise-induced mitochondrial biogenesis and angiogenesis in trained skeletal muscle. These findings add to existing data demonstrating that in addition to the amount of total work done, the interval/continuous nature of a training session appears to have little effect on the observed cell signalling response. Whilst these data have implications for exercise prescription and implicate exercise intensity as the key driver in regulating adaptation to low-volume exercise, training studies employing similar ‘all-out’ exercise protocols are warranted to investigate their effects on structural re-modelling, whole body metabolism and exercise performance.

## Abbreviations

AMP: Adenosine monophosphate
AMPK: Adenosine monophosphate-activated protein kinase
ACC: Phospho-acetyl-CoA carboxylase
ATP: Adenosine triphosphate
CON: Continuous-based ‘all-out’ cycling protocol
eNOS: Endothelial nitric oxide synthase
HIF-1α: Hypoxia inducible factor 1 alpha subunit
INT: Interval-based ‘all-out’ cycling protocol
MAP: Maximal aerobic power
MPO: Mean power output
MMP-9: Matrix metalloproteinase 9
mRNA: Messenger RNA
PGC-1α: Peroxisome proliferator-activated γ receptor coactivator 1 alpha subunit
PPO: Peak power output
RNA: Ribonucleic acid
RT-PCR: Real time polymerase chain reaction
SIT: Sprint interval training
SE: Standard error
VEGF: Vascular endothelial growth factor
$\dot{V}{\text{O}}_{{2{\text{peak}}}}$: Peak oxygen uptake
